# Non-Canonical Wnt Signaling and N-Cadherin Related β-Catenin Signaling Play a Role in Mechanically Induced Osteogenic Cell Fate

**DOI:** 10.1371/journal.pone.0005388

**Published:** 2009-04-29

**Authors:** Emily J. Arnsdorf, Padmaja Tummala, Christopher R. Jacobs

**Affiliations:** 1 Bone and Joint R&D Center, VA Palo Alto Health Care System, Palo Alto, California, United States of America; 2 Department of Bioengineering, Stanford University, Stanford, California, United States of America; 3 Department of Mechanical Engineering, Stanford University, Stanford, California, United States of America; 4 Department of Biomedical Engineering, Columbia University, New York, New York, United States of America; UT MD Anderson Cancer Center, United States of America

## Abstract

**Background:**

Understanding how the mechanical microenvironment influences cell fate, and more importantly, by what molecular mechanisms, will enhance not only the knowledge of mesenchymal stem cell biology but also the field of regenerative medicine. Mechanical stimuli, specifically loading induced oscillatory fluid flow, plays a vital role in promoting healthy bone development, homeostasis and morphology. Recent studies suggest that such loading induced fluid flow has the potential to regulate osteogenic differentiation via the upregulation of multiple osteogenic genes; however, the molecular mechanisms involved in the transduction of a physical signal into altered cell fate have yet to be determined.

**Methods and Principal Findings:**

Using immuno-staining, western blot analysis and luciferase assays, we demonstrate the oscillatory fluid flow regulates β-catenin nuclear translocation and gene transcription. Additionally, real time RT-PCR analysis suggests that flow induces Wnt5a and Ror2 upregulation, both of which are essential for activating the small GTPase, RhoA, upon flow exposure. Furthermore, although β-catenin phosphorylation is not altered by flow, its association with N-cadherin is, indicating that flow-induced β-catenin signaling is initiated by adherens junction signaling.

**Conclusion:**

We propose that the mechanical microenvironment of bone has the potential to regulate osteogenic differentiation by initiating multiple key molecular pathways that are essential for such lineage commitment. Specifically, non-canonical Wnt5a signaling involving Ror2 and RhoA as well as N-cadherin mediated β-catenin signaling are necessary for mechanically induced osteogenic differentiation.

## Introduction

Mechanical signals play a vital role in promoting healthy bone homeostasis and morphology [1]–[7]. Specifically, a lack of mechanical stimuli results in tissue loss similar to that of osteoporosis [8]–[10]. Alternatively, increased loading stimulates an increase in bone density and alterations in morphology [11]. Recent studies have suggested that cells within fracture sites have the potential to induce mesenchymal stem cell (MSC) migration to sites of wound healing indicating that progenitor cells may migrate into bone and initiate osteogenic lineage commitment based on the microenvironmental cues within the bone tissue, itself [12]. Several computational and *in vivo* models of bone loading have suggested that loading induces a dynamic flow profile of interstitial fluid through the bone matrix [13]–[15]. Such loading induced fluid flow initiates activity in mature osteoblasts and osteocytes that would promote healthy bone homeostasis [16]–[21], and recently it was shown to be a potent signal in guiding osteogenic lineage commitment of MSCs by inducing Runx2, Osteopontin and Osteocalcin upregulation [22], [23]. However, in addition to understanding that physical signals regulate cell fate, it is necessary to understand how they guide it, specifically by what molecular mechanisms.

Although there are multiple molecular pathways involved in osteogenic differentiation, the established role of the Wnt family of proteins in bone development and homeostasis make it an ideal candidate to be involved in flow induced osteogenic lineage commitment [24]–[37]. Wnts are a family of 19 secreted proteins that can act through two classically defined pathways: the canonical and the non-canonical pathway [38]–[41]. Both pathways are transduced through the Frizzled family of receptors along with a co-receptor (LDL proteins, LRP5/6 for canonical and receptor-type kinases, Ror1 and Ror2, for non-canonical signaling) [42]. In the current model of canonical Wnt signaling, the absence of Wnt ligand enables a protein complex comprised of glycongen-synthase kinase 3β (GSK3β), adenomatous polyposis coli (APC), and Axin [26], [43] to sequester β-catenin. Once sequestered to the complex, GSK3β phosphorylates β-catenin at Ser-33/Ser-37/Thr-41 sites [44], [45] targeting it for ubiquitinylation and ultimately proteosomal degradation [43]. However, when Wnts bind to the co-receptor complex and initiate canonical signaling, GSK3β activity is inhibited, which enables β-catenin stabilization, cytoplasmic accumulation and eventual translocation to the nucleus [26], [42]. Once inside the nucleus, β-catenin associates with members of the T-cell factor (TCF) and lymphoid enhancer factor (LEF) transcription factors resulting in increased expression of target genes [26], [42], [46]–[50].

In the last decade, canonical Wnt signaling has become a central focus in studying the promotion of healthy bone tissue as well as a target for potential therapeutic applications. Primarily, loss of function mutations of the canonical receptor, LRP5, result in osteopenia or familial cases of osteoporosis, while gain of function mutations result in an autosomal high bone density trait [26], [50]–[52]. Additionally, β-catenin signaling promotes osteogenic differentiation in MSCs downstream of hedgehog and BMP2 signaling [24], [28], [53], [54]. Furthermore, TCF has an active binding site in the Runx2 promoter indicating that the β-catenin/TCF/LEF complex has the potential to directly regulate Runx2 expression [55]. In contrast, several recent studies suggest that canonical Wnt signaling may actually promote stem cell renewal, and inhibit osteogenic differentiation [25], [26], [56]. Examining wound healing in mice with the same gain of function mutation in LRP5, Kim and colleagues showed that at the injury site osteoprogenitor cells maintained their proliferative state and have delayed osteoblastic differentiation resulting in an 84% reduction in injury-induced bone regeneration compared with controls [56]. Furthermore, *in vitro* LRP5 over-expression as well as canonical Wnt3a signaling both resulted in significant proliferation of MSCs [25], [26]. In fact, a recent study by Zhang and colleagues demonstrated that the osteoblast-specific transcription factor, Osterix, which is necessary for osteogenic differentiation, inhibits canonical Wnt signaling upon expression hindering Wnt induced proliferation [57]. Interestingly, canonical Wnt signaling appears to enhance MSC self-renewal and hinder osteogenic differentiation; nonetheless, β-catenin signaling is a necessary signal for osteogenic lineage commitment.

Although canonical Wnt signaling via β-catenin translocation may promote differentiation in specific conditions, it should be noted that β-catenin translocation to the nucleus may also be regulated by cadherin signaling [58]–[61]. Cadherins comprise a family of calcium-dependent glycoproteins that function in mediating cell-cell adhesion and are anchored to the actin cytoskeleton through either β-catenin/α-catenin or plakoglobin (γ-catenin)/α-catenin complexes [62]. Junctional disassembly of cadherin-catenin complexes result in an increase in cytoplasmic β-catenin that then has the potential to translocate to the nucleus in a manner similar to canonical Wnt signaling. However, cadherin signaling has not yet been shown to be involved in osteogenic differentiation and moreover, it is unclear if either of these mechanisms are involved in the mechanical regulation of cell fate.

The non-canonical Wnt signaling pathway, which does not involve β-catenin, is transduced through Frizzled and Ror1 or Ror2 co-receptor complexes and has the potential to initiate the RhoA, JNK and calcium signaling pathways among others [42], [63]. Although less studied relative to canonical Wnt signaling, aspects of non-canonical Wnt signaling have been shown to play essential roles in guiding MSC fate. Particularly, the activation of the small GTPase RhoA and its direct effector protein, ROCK, have vital roles in MSC fate commitments between the adipogenic, chondrogenic and osteogenic lineage pathways [23], [64]–[66] and have been demonstrated to have a necessary role in mechanically induced osteogenic differentiation [23]. Furthermore, over expression of the receptor protein, Ror2, results in Runx2 and Osterix upregulation as well as *ex vivo* mineralization [67], [68]. Finally, Wnt5a, has been shown to be important in multiple aspects of development as well as MSC lineage commitment to the osteoblast cell fate [25], [26], [69]. In fact, non-canonical Wnt5a has an antagonistic functional relationship with canonical Wnt3a induced stem cell renewal, making it a prime candidate in flow induced Runx2 expression [25], [26], [70].

In this study, we examine the potential of mechanical stimuli to regulate classically defined canonical and non-canonical signaling pathways in C3H10T1/2 murine MSCs by investigating the induction of β-catenin translocation and expression of Wnt associated proteins with fluid flow. Additionally, we determine if β-catenin and Wnt5a signaling are involved in mechanically induced osteogenic differentiation via Runx2 upregulation. Furthermore, we investigate Wnt5a's potential to activate RhoA, a possible non-canonical effector shown to be necessary for flow induced Runx2 expression, and determine if the tyrosine receptor-type kinase, Ror2, is involved. Finally, we examine the molecular mechanism governing β-catenin signaling and translocation to the nucleus. We propose that non-canonical Wnt5a signaling involving Ror2 and RhoA as well as N-cadherin mediated β-catenin signaling are necessary for mechanically induced osteogenic differentiation.

## Methods

### Cell Culture

C3H10T1/2 mesenchymal stem cells (ATCC) were cultured in Eagle's Basal medium (BME) with 2 mM L-glutamine (Sigma). Media was adjusted to contain 2.2 g/L sodium bicarbonate and supplemented with 10% fetal bovine serum (Hyclone), 1% penicillin and streptomycin (Invitrogen). Cells were maintained at 37°C and 5% CO_2_.

### siRNA transfection and pharmacological agents

To prevent Wnt5a or Ror2 signaling, 0.20 µM siRNA targeting Wnt5a (Santa Cruz Biotechnology, Cat # sc-41113), 0.20 µM siRNA for Ror2 (Invitrogen, 5′- AAU CUU CCA CYY CAC CUG C- 3′) or 0.20 µM scrambled control siRNA (Santa Cruz biotechnology, Cat # sc-37007) were transfected into cells using 10 µl of XtremeGene (Roche Molecular Systems) and OptiMEM (Invitrogen) media. After 4 hours of incubation, media was put on cells and flow experiments were performed 48 hours later. Neither the scrambled siRNA, Wnt5a siRNA, nor Ror2 siRNA had any significant effect on overall cellular morphology. To inhibit β-catenin signaling, cells were incubated with 15 µg/ml of endostatin (Sigma) for 24 hours prior to flow.

### Oscillatory fluid flow

Cells were subcultured on fibronectin coated glass slides (76×35×1 mm) at 140,000 cells/slide. Fluid flow was applied to cells that were 80–90% confluent at the time of experimentation. A previously described fluid flow device was used to deliver oscillatory fluid flow to C3H10T1/2 progenitor cells [71]. In brief, oscillatory flow was driven by a Hamilton glass syringe in series with rigid walled tubing and a parallel plate flow chamber. The syringe was mounted in and driven by a mechanical loading device. The flow rate was monitored with an ultrasonic flow meter (Transonic Systems Inc.) and was selected to yield peak shear stresses of 1.0 Pa (10 dynes/cm^2^). The dynamic flow profile was sinusoidal at a frequency of 1 Hz. For β-catenin studies, cells were exposed to flow for 35 minutes and lysed immediately. For Wnt expression studies, cells were exposed to 15, 30 or 45 minutes of flow and lysed immediately. For RhoA assays, cells were exposed to 1 hour of flow and lysed immediately. For Runx2 expression, cells were exposed to 1 hour of flow followed by a 30 minute incubation in fresh BME growth media, then lysed.

### RNA isolation and Real Time RT-PCR

Cells were lysed after flow exposure and total RNA was isolated using Tri-Reagent (Sigma). The 260/280 absorbance ratio was measured for verification of the purity and concentration of the RNA. Reverse transcription was completed using GeneAMP RNA PCR Core kit (Applied Biosystems) with 0.75 µg of RNA. Analysis by quantitative real-time RT-PCR (Perkin Elmer Prism 7900, Applied Biosystems) was conducted using Taqman PCR Master Mix and a 20× 18S primer and probe (Applied Biosystems), or by using SYBER green PCR master mix with primers and probes developed by Operon technologies for Runx2, Wnt3a, Wnt5a, Ror2. The primer sequences were: Runx2 Forward 5′-AGA AGG CAC AGA CAG AAG CTT GA- 3′; Runx2 Reverse 5′- AGG AAT GCG CCC TAA ATC ACT-3′; Wnt5a Forward 5′- CTC CTT CGC CCA GGT TGT TAT AG -3′; Wnt5a Reverse 5′- TGT CTT CGC ACC TTC TCC AAT G -3′; Wnt3a Forward 5′- ATA GCC TGC ATC CGC TCT GA -3′; Wnt3a Reverse 5′- TGG TGA CCA TTG CCT CAA CA -3′; Ror2 Forward 5′-TGG AAC TGT GTG ACG TAC CC -3′; Ror2 Reverse 5′-GCG AGG CCA TCA GCT G -3′. Each sample was analyzed in triplicate. Gene expression levels were normalized against 18S rRNA assessed from the same sample tube.

### Immuno-staining

To detect β-catenin translocation, cells were exposed to oscillatory fluid flow for 35 minutes, then immediately fixed in 3.7% formaldehyde and membranes were removed with 0.1% Triton-X in PBS. Cells were incubated with 1∶100 monoclonal mouse anti-β-catenin antibody (BD biosciences, Cat # 610153) and 1∶100 donkey IgG in primary blocking solution (PBS, 1% BSA, 0.1% NP-40) overnight. Cells were then washed with 1× PBS for 10 minutes for a total of 3 times and incubated with FITC-conjugated donkey anti-mouse IgG (Jackson ImmunoResearch Labs) for 30 minutes. Cells were washed 3 times for 5 minutes in PBS. Cover slips were mounted on the slides using 35 µl of Vectashield mounting medium (Vector Laboratories) and sealed with nail polish.

### RhoA-GTP assay

RhoA-GTP was measured by pull-down assay (adapted from [64], [72]). Immediately following exposure to 1 hour of oscillatory fluid flow, cells (8 slides per sample) were rinsed with ice-cold TBS, then lysed in 200 µl of lysis buffer (50 mM Tris (pH 7.2)) (Quality Biologicals), 1%Triton X-100, 0.5% sodium deoxycholate, 0.1% sodium dodecyl sulfate, 500 mM NaCl, 10 mM MgCl_2_, 10 µg/ml aprotinin/leupeptin, and 1 mM PMSF (Sigma)) at 4°C. Samples were centrifuged for 3 minutes at 3000 rcf at 4°C. 30 µl of supernatant was removed and used to determine total RhoA; the remaining volume of supernatant was incubated with rhotekin binding domain-beads (Millipore) for 45 min at 4°C. After incubation, samples were centrifuged for 3 minutes at 3000 rcf, and washed three times using IP buffer (10 mM Tris-HCl at pH 7.5, 1% Triton X-100, 0.5% NP-40, 150 mM NaCl, 2 mM CaCl_2_, 0.1 mM sodium orthovanadate, 10 µg/ml aprotinin/leupeptin, and 1 mM PMSF (Sigma)), and suspended in SDS-PAGE buffer. RhoA was assayed by Western blot.

### Protein isolation

To determine β-catenin nuclear localization, phosphorylation, and association with N-cadherin, cells were exposed to 35 minutes of flow. For β-catenin translocation, after the cessation of flow, cells (1 slide per sample) were immediately trypsinized and centrifuged at 5000 rcf for 5 minutes and supernatant was removed. Nuclear and cytoplasmic protein were isolated using NE-PER Nuclear and Cytoplasmic Extraction Reagents (Pierce Biotechnology). The kit protocol was followed with the addition of proteinase inhibitor, PMSF and sodium orthovate to the reagents: CER I and NER.

To determine the effects of oscillatory fluid flow on N-cadherin and β-catenin association, cells were lysed immediately following the cessation of flow (1 slide per sample) using 100 µl of lysis buffer. Samples were sonicated for 5 seconds and centrifuged at 3000 rcf for 3 minutes. The supernatant was incubated with 25 µl Protein G sepharose beads (Amersham Pharmacia) for 15 min and centrifuged for 2 min at 14,000 rcf. The supernatant was incubated with 2.5 µl of anti-β-catenin antibody for 30 min, and then 50 µl of Protein G sepharose beads was added and incubated for 1 hour. The beads were washed four times with IP buffer (10 mM Tris-HCl at pH 7.5, 1% Triton X-100, 0.5% NP-40, 150 mM NaCl, 2 mM CaCl_2_, 0.1 mM sodium orthovanadate, 10 µg/ml aprotinin/leupeptin, and 1 mM PMSF (Sigma)) and resuspended in SDS page buffer.

To determine phosphorylated β-catenin levels with flow, cells (2 slides per sample) were lysed immediately after 35 minutes of flow in 100 µl of lysis buffer, sonicated and centrifuged for 3 minutes at 3000 rcf. Supernatants were used for protein quantification.

### Western Blot analysis

Protein concentration in the supernatant was determined using a Bradford protein assay kit (Bio-Rad Laboratories, Inc.). Suspended samples were electrophoresed through NuPAGE 4–12% Bis-Tris polyacrylamide gels (Invitrogen) and were transferred electrophoretically onto nitrocellulose membranes in blocking buffer (Pierce). After washing, membranes were incubated with primary antibody overnight at 4°C under gentle rocking. Incubation with a HRP-conjugated anti-goat/mouse IgG (1∶2000 dilution) was carried out for 1 hour at room temperature under gentle rocking. Signals were visualized using a chemiluminescent ECL substrate (GE Healthcare). Densitometric analysis was performed using ImageQuant software. Protein levels were normalized using total RhoA (for RhoA assays), beta actin (for β-catenin assays) or β-catenin (for β-catenin phosphorylation and N cadherin co-immunoprecipitation).

Antibodies used were: β-catenin (1∶2000, BD Biosciences, Cat # 610154), N-cadherin (1∶5000, BD Biosciences, Cat # 610920), RhoA (1∶2000, Santa Cruz Biotechnology, Cat. # sc-418), Serine 33 Phosphorylated β-catenin (1∶200, Santa Cruz Biotechnology, Cat. # sc-16743-R).

### Luciferase detection

Luciferase detection was determined with the Dual Light System (Applied Biosystems). Mouse L cells transfected with TOPflash (described by Mikels and Nusse [70]) were exposed to 35 minutes of oscillatory and immediately lysed in 250 µl Lysis Solution with 0.5 mM Dithiothreitol (Sigma). Luciferase assays were performed using Dual-Light reporter gene assay system (Applied Biosystems). Relative luciferase units were measured and normalized against β-galactosidase activity.

### Data Analysis

Data are expressed as mean±standard error. A student T-test was used to compare control cells and cells exposed to fluid flow. A p<0.05 was considered significant.

## Results

### Oscillatory fluid flow induces β-catenin translocation and TCF/LEF-dependent gene transcription

**Figure 1:**
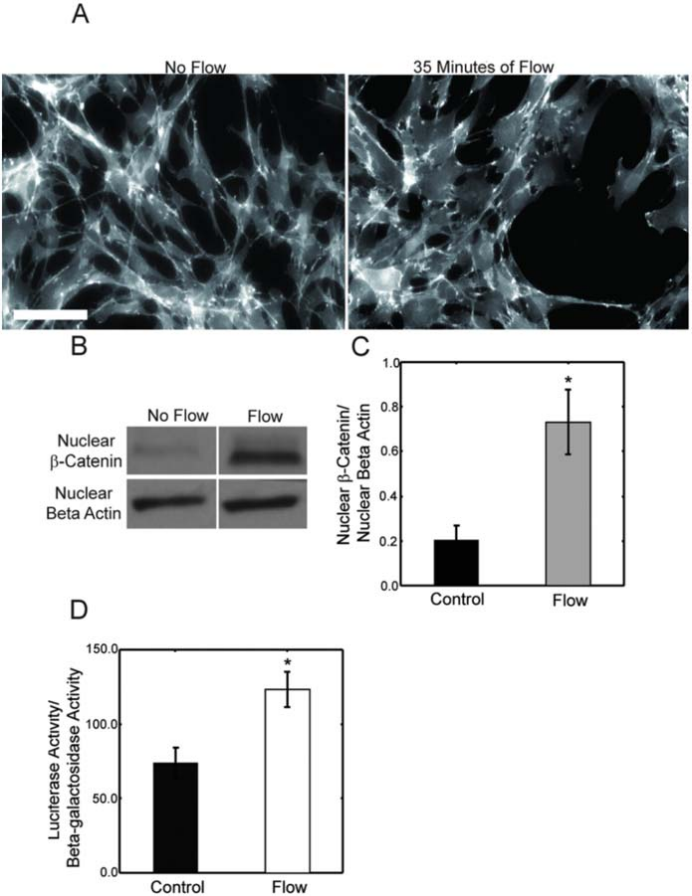
Oscillatory fluid flow induces β-catenin nuclear translocation and initiation of TCF/LEF associated gene transcription. (A) Immuno-staining of control and cells exposed to oscillatory fluid flow illustrate that 35 minutes of mechanical stimulation results in an increase in β-catenin localization to the nucleus. (B) To validate β-catenin translocation with flow, nuclear protein was isolated and western blot analysis was used to demonstrate that there is a 3.5±0.9-fold (p<0.05) increase in nuclear β-catenin in mechanically stimulated cells versus control. (C) To determine β-catenin/TCF/LEF transcription, TOPflash transfected mouse L cells were exposed to 35 minutes of oscillatory fluid flow. A dual light luciferase assay was used to determine that mechanical stimulation induced a 2.0±0.4-fold increase (p<0.05) in luciferase activity indicating an increase in β-catenin/TCF/LEF transcription. Scale bar = 100 µm. (Error bars: SEM (n≥4)).

Immuno-staining of control cells and cells that had been exposed to 35 minutes of oscillatory fluid flow indicated that, with mechanical stimulation, there is a greater amount of β-catenin localized to the nucleus (Fig. 1A). To validate that oscillatory fluid flow indeed stimulates β-catenin translocation, cells were again exposed to 35 minutes of flow and nuclear protein was isolated. Western blot analysis demonstrated that nuclear β-catenin levels were increased 3.5±0.9-fold in cells exposed to flow versus control cells (Fig. 1B, C). For a further validation, we exposed murine fibroblastic L-cells transfected with TOPflash to oscillatory fluid flow. Cells transfected with TOPflash, luminesce upon β-catenin mediated TCF/LEF-dependant gene transcription, making it a widely used Wnt signaling reporter. An exposure to 35 minutes of oscillatory fluid flow elicited a significant 2.0±0.4-fold increase in luciferase activity with flow exposure compared to control cells, indicating that β-catenin does translocate to the nucleus and initiate TCF/LEF dependent transcription in response to flow (Fig. 1D).

### Oscillatory fluid flow initiates the upregulation of several Wnt associated proteins

**Figure 2:**
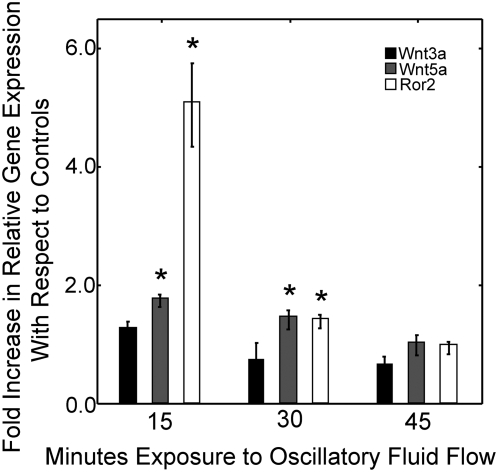
Non-canonical related Wnt proteins are regulated by oscillatory fluid flow. To determine if Wnt expression was regulated by mechanical stimulation, MSCs were exposed to 15, 30 and 45 minutes of flow and lysed immediately. The canonical Wnt, Wnt3a was not affected by flow exposure. However, non-canonical associated Wnt5a and tyrosine kinase receptor, Ror2, were significantly upregulated with 15 and 30 minutes of flow. Wnt5a expression was increased 1.8±0.1-fold (p<0.01) in cells exposed to 15 minutes of flow versus control cells and was maintained after 30 minutes of flow to be 1.5±0.2-fold (p<0.05). Ror2 expression was increased 5.1±0.7-fold (p<0.01) in cells after 15 minutes of oscillatory fluid flow and 1.4±0.1-fold (p<0.05) after 30 minutes. C3H10T1/2 MSCs did not express canonical Wnt7b or Wnt10b. (Error bars: SEM (n≥10)).

Based on our finding that β-catenin signaling is induced by oscillatory fluid flow, we next wanted to determine if oscillatory fluid flow regulated the expression profile of several known Wnt proteins associated with osteogenic differentiation or stem cell renewal that may initiate β-catenin nuclear translocation (Fig. 2). Cells were exposed to 15, 30 and 45 minutes of flow and gene expression was determined using real time RT-PCR. Wnt5a, a Wnt with the potential to activate both canonical and non-canonical signaling [70], was significantly upregulated by 1.8±0.1-fold in cells exposed to flow for 15 minutes and by 1.5±0.2-fold in cells exposed to 30 minutes of flow compared to control cells. There was no significant difference in Wnt5a expression between control cells and cells exposed to 45 minutes of flow. Additionally, the non-canonical tyrosine kinase receptor, Ror2, which itself has a role in Runx2 and Osterix regulation [67], [68], was significantly upregulated in cells exposed to 15 and 30 minutes of flow relative to controls by 5.1±0.7-fold and 1.4±0.1-fold, respectively. Wnt3a expression, which has been associated with MSC self-renewal, was not affected by flow exposure at any time points. C3H10T1/2 cells did not express canonical Wnt7b or Wnt10b.

### Wnt5a signaling and β-catenin signaling are necessary for flow induced osteogenic differentiation

**Figure 3:**
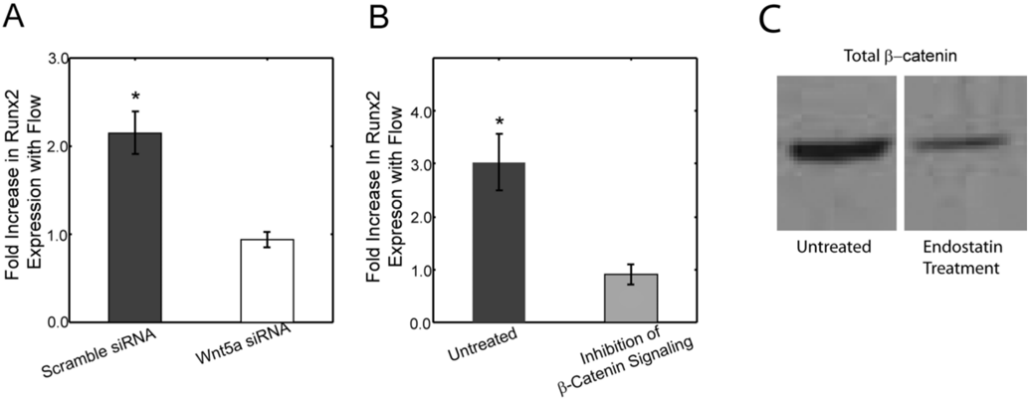
Wnt5a and β-catenin signaling are both necessary for flow induced Runx2 upregulation. (A) Flow induced Runx2 expression was upregulated 2.2±0.2-fold in scrambled siRNA treated cells exposed to flow verses scrambled siRNA control cells. This fold increase was significantly different (p<0.05) than Wnt5a siRNA treated cells, in which the flow induced Runx2 expression was abrogated. (B) The fold change in Runx2 expression with flow was significantly different between untreated cells and cells with inhibited β-catenin signaling via endostatin treatment (p<0.01). Untreated cells exposed to oscillatory fluid flow had a 2.8±0.5-fold increase in Runx2 expression over control cells; while endostatin treated cells resulted in no difference between flowed and control cells. (C) Western blot analysis demonstrates that there is a significant decrease in β-catenin levels after a 24 hour incubation with endostatin. (Error bars: SEM (n≥6)).

To determine the role of Wnt5a and β-catenin signaling in flow induced osteogenic lineage commitment, cells were treated with several inhibitory factors, exposed to fluid flow, and Runx2 expression was compared to that of control cells. Primarily, we interrupted Wnt5a signaling using siRNA technology that resulted in a significant 55% decrease in Wnt5a mRNA expression levels. Inhibiting Wnt5a signaling via siRNA significantly reduced the cell's ability to upregulate Runx2 in the presence of mechanical stimulation. Specifically, scrambled siRNA treated cells exposed to fluid flow had a 2.2±0.2-fold increase in Runx2 expression compared to control cells. However, Wnt5a siRNA treated cells had no difference in Runx2 expression between flow-exposed and control cells (Fig. 3A). To investigate the role of β-catenin, cells were incubated with endostatin, which stimulates the degradation of β-catenin and inhibits nuclear translocation upon flow exposure [73]. Endostatin treatment resulted in a significant decrease in flow-induced Runx2 expression compared to untreated cells (Fig. 3B). Untreated cells exposed to oscillatory fluid flow had a 2.8±0.5-fold increase in Runx2 expression over control cells; while endostatin treated cells had no difference in Runx2 expression between flowed and control cells.

### Wnt5a and Ror2 are necessary for flow induced RhoA activation, but Wnt5a does not regulate β-catenin signaling

**Figure 4:**
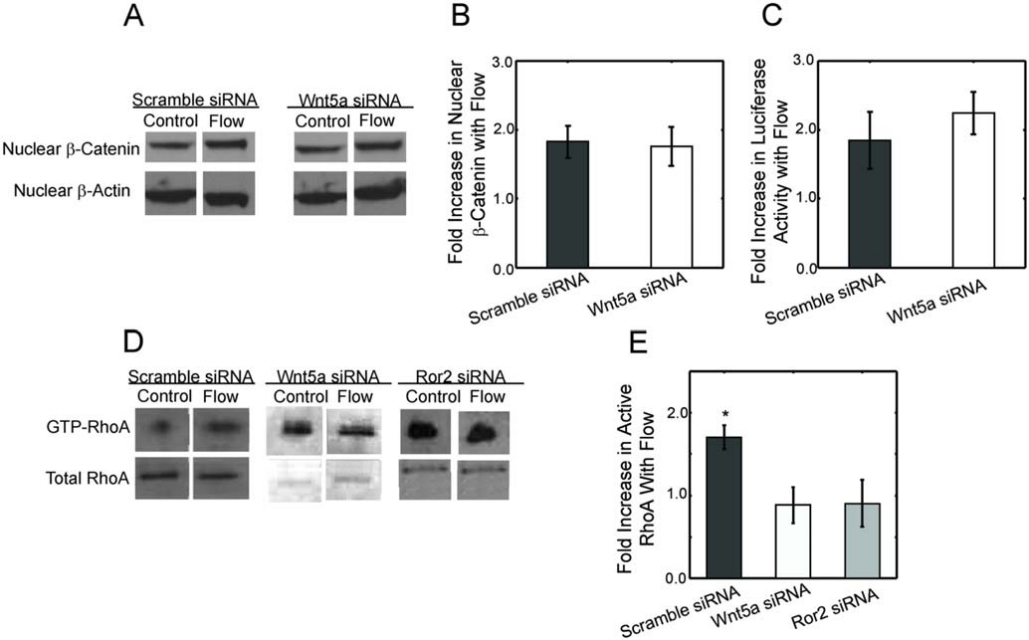
Wnt5a and Ror2 are necessary for flow-induced RhoA activation, but do not effect β-catenin translocation. (A) Western blots of nuclear β-catenin indicate that both scrambled and Wnt5a siRNA treated cells maintained their potential to initiate β-catenin signaling with flow. (B) Analysis of the western blots demonstrates that scrambled and Wnt5a siRNA treated cells had a 1.9±0.2-fold and 1.8±0.3-fold increase in nuclear β-catenin with flow, respectively. (C) β-catenin/TCF/LEF transcription of downstream genes was also maintained in both scramble and Wnt5a treated cells with a 1.8±0.4-fold and 2.2±0.3-fold increase in luciferase activity, respectively, indicating that Wnt5a is not necessary for mechanically induced β-catenin signaling. (D) Western blots were used to assay Rho activation in response to flow in scrambled, Wnt5a and Ror2 siRNA treated cells. (E) Analysis of the blots indicates that the 1.7±0.1-fold increase observed in scrambled treated cells is significantly greater than Wnt5a (p<0.01) and Ror2 (p<0.05) siRNA treated cells, both of which lost flow-induced RhoA activation. (Error bars: SEM (n≥4)).

Given that Wnt5a and β-catenin signaling are necessary for flow-induced osteogenic differentiation, and that Wnt5a has the potential to induce both the canonical and non-canonical pathways, we next investigated whether flow-induced Wnt5 signaling was activating the canonical or non-canonical pathways (Fig. 4). To determine if Wnt5a plays a role in flow-induced β-catenin signaling, both C3H10T1/2 MSCs and TOPflash transfected mouse L fibroblast cells were treated with scrambled or Wnt5a siRNA and exposed to flow. Western Blot analysis of nuclear β-catenin indicated that both scrambled and Wnt5a treated cells maintained the potential to induce β-catenin nuclear translocation with flow by a 1.9±0.2-fold and 1.8±0.3-fold, respectively (Fig. 4A and 4B). Furthermore, cells also maintained the potential to initiate β-catenin/TCF/LEF transcription given that flow induced luciferase activity was maintained in both scrambled and Wnt5a treated cells with a 1.8±0.4-fold and 2.2±0.3-fold increase, respectively (Fig. 4C).

Given that β-catenin signaling was not the target of Wnt5a, we next determined if Wnt5a signaling was necessary for flow induced RhoA activation. Furthermore, we also used Ror2 siRNA, which significantly decreased Ror2 mRNA expression, to determine if this receptor was, in fact, necessary. For these studies cells treated with scrambled, Wnt5a, and Ror2 siRNA were exposed to oscillatory fluid flow and a pull down assay utilizing agarose beads with the rhotekin binding domain enabling the isolation of active RhoA was used to isolate GTP-bound RhoA. Analysis of the blots indicates that the 1.7±0.1-fold increase in active RhoA observed in scrambled treated cells was significantly greater than Wnt5a and Ror2 siRNA treated cells (Fig. 4D). Both of these treatments resulted in attenuated flow-induced RhoA activation (Fig. 4E) suggesting that both Wnt5a and Ror2 signaling are necessary for mechanically stimulated RhoA activation, which ultimately results in Runx2 upregulated [23].

### β-catenin Signaling is mediated via cadherin-catenin signaling

**Figure 5:**
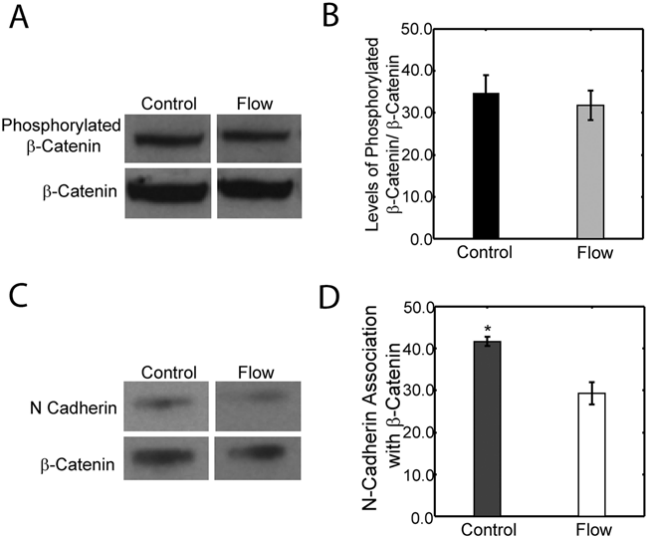
Flow-induced β-catenin signaling may be mediated by cadherin signaling rather than canonical Wnt signaling. (A) Western blots were used to determine the level of phosphorylated β-catenin in cells exposed to oscillatory fluid flow versus controls. (B) Analysis of the western blot indicates that there is no significant difference between control and experimental cells in the level of phosphorylated β-catenin. (C) A western blot was also used to assay N-cadherin association with β-catenin as a function of mechanical stimulation. (D) Analysis of the western blot demonstrates that there is a significant 30% decrease in β-catenin/N-cadherin association with exposure to 35 minutes of oscillatory fluid flow (p<0.01). (Error bars: SEM (n≥4)).

To determine the mechanism inducing β-catenin signaling, we examined both β-catenin phosphorylation with flow, mediated by canonical Wnts, and N-cadherin/β-catenin association, mediated by cadherin signaling (Fig. 5). Western blot analysis was used to determine the level of phosphorylated β-catenin in cells exposed to oscillatory fluid flow versus control cells; there was no significant difference in phosphorylated β-catenin levels between control and experimental cells (Fig. 5A, B). To determine N-cadherin and β-catenin association with flow, β-catenin was immuno-precipitated and a western blot was used to analyze N-cadherin association (Fig. 5C). Analysis of the western blots demonstrated that 35 minutes of mechanical stimulation induced a significant 30% decrease in β-catenin/N-cadherin association suggesting that adherens junctions may be a mechanosensory that plays a potent role in mechanically stimulated cell fate (Fig. 5D).

## Discussion

**Figure 6:**
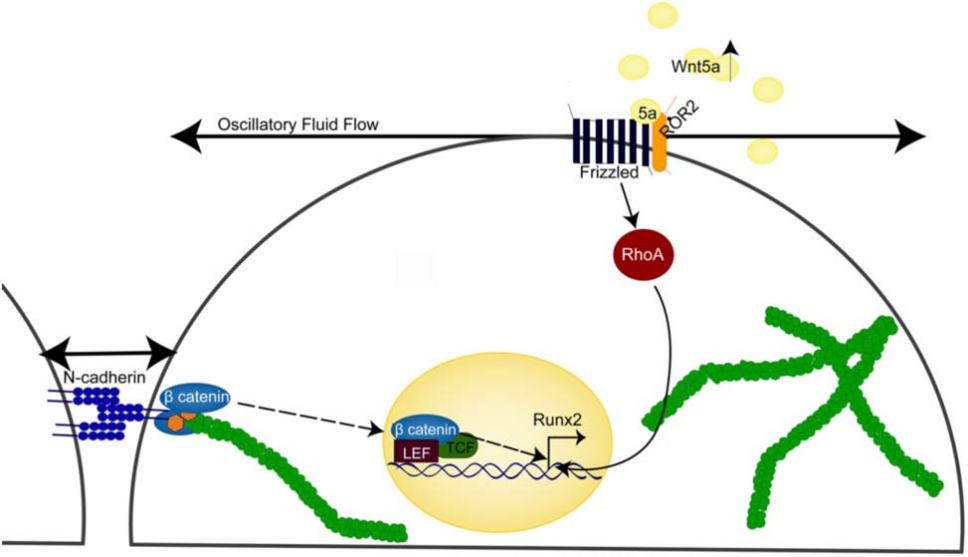
A schematic diagram of potential signaling mechanisms involved in mechanically stimulated osteogenic differentiation. Oscillatory fluid flow, a potent mechanical signal within the microenvironment of bone has the potential to regulate non-canonical Wnt5a and β-catenin signaling pathways in MSCs, both of which are essential for fluid flow induced osteogenic lineage commitment via Runx2 upregulation. Furthermore, Wnt5a signals through Ror2 to activate RhoA, a small GTPase that is necessary and sufficient for osteogenic differentiation. Finally, flow induced β-catenin signaling appears to be mediated by alterations in N-cadherin/β-catenin association indicating that adherens junctions may be involved in the transduction of a mechanical signaling into a cell fate decision.

The mechanical microenvironment of bone has the potential to regulate osteogenic lineage commitment by initiating multiple key molecular pathways that are essential for cell fate commitment. Understanding what molecular mechanisms are involved in lineage commitment will not only enhance the current understanding of stem cell biology but also the field of regenerative medicine. In this study, we show that mechanical stimulation is a potent signal that initiates both non-canonical Wnt signaling and β-catenin signaling. Additionally, flow induced β-catenin signaling initiates the transcription of β-catenin/TCF/LEF downstream genes, including Runx2. Furthermore, flow upregulates Wnt5a and Ror2, both of which are necessary for flow induced RhoA activation and ultimately, Runx2 expression [23]. Finally, our results suggest that β-catenin signaling may be regulated by adherens junctions. Specifically, we found that oscillatory fluid flow alters the association of N-cadherin with β-catenin, increasing the cytoplasmic pool of β-catenin without alterations in β-catenin phosphorylation. We propose that both Wnt5a non-canonical signaling via Ror2 and RhoA, and β-catenin signaling are necessary for flow induced osteogenic lineage commitment indicating multiple pathways must be initiated for alterations in cell fate (Fig. 6).

Our study is the first to suggest that Wnt5a is upregulated by mechanical stimuli and is necessary for flow induced osteogenic lineage commitment. These results are consistent with previous reports that have demonstrated Wnt5a is important in MSC osteogenic differentiation [25], [26]. Additionally we demonstrate that inhibiting Wnt5a signaling as well as Ror2 expression abrogates flow induced RhoA activation, a protein that has been shown to be both necessary and sufficient in initiating osteogenic differentiation [23], [64]. One recent study illustrated that Wnt5a has the potential to induce osteogenic differentiation in cells cultured on tissue culture plastic, but not in suspension [25]. This difference in response depending on culture conditions may be due to the effect of RhoA on the cytoskeleton. RhoA activation ultimately results in generation of isometric tension within the actin cytoskeleton, which itself, has been shown to be important for osteogenic differentiation. However, in suspension, cells remain in a more rounded morphological state that lacks cellular adhesions and thus, may inhibit the cell's ability to generate tension within the actin cytoskeleton. Taken together, therefore, Wnt5a's capability to activate RhoA, ultimately resulting in lineage commitment appears to be dependent on cell shape or cell adhesion.

Oscillatory fluid flow induces β-catenin nuclear translocation and the initiation of TCF/LEF/β-catenin transcription, which is necessary for flow induced Runx2 expression. However, oscillatory flow does not appear to regulate canonical Wnt3a, and other Wnt proteins associated with osteogenesis were not expressed in C3H10T/2 MSCs. Furthermore, β-catenin phosphorylation is not altered upon flow exposure suggesting that flow-induced β-catenin signaling is not due to canonical Wnt signaling. Interestingly, our results show that the association between N-cadherin and β-catenin is decreased upon mechanical stimulation. This suggests the possibility that flow-induced liberation of β-catenin in adherens junctions or cell-cell contacts may be the first step in enabling β-catenin to accumulate in the cytoplasm and ultimately translocate to the nucleus to initiate transcription in response to mechanical stimulation. Our findings support previous work conducted by Norvell and colleagues demonstrating that upon mechanical stimulation with unidirectional fluid flow, β-catenin is also induced to translocate to the nucleus and initiate transcription of Cox2, another osteogenic gene, in MC3T3-E1 pre-osteoblast cells [74]. Taken together, these results suggest that β-catenin is an important pathway in increased osteogenic activity in response to mechanical stimulation [75].

Additionally, we show that both β-catenin signaling, which may be mediated by cadherin signaling, and Wnt5a/RhoA signaling is necessary for flow induced osteogenic differentiation. This is indicative that multiple intracellular pathways must be initiated for lineage commitment to occur. In fact, studies indicate that stabilized β-catenin has the potential to induce Runx2 and Osteopontin expression, but it does not induce Osteocalcin expression [76]–[78] suggesting that while it may be able to initiate differentiation, other molecular pathways must be present to enable full osteogenic lineage commitment of stem cells. Although it has not yet been established to be involved in osteogenic differentiation, N-cadherin has been shown to play a significant role in chondrogenic differentiation. Specifically, N-cadherin mediated cell-cell interactions in micromass cultures of mesenchymal cells induced chondrogenesis both *in vitro* and in the intact limb bud *in vivo* [60], [79], [80]. Furthermore, N-cadherin has the potential to inhibit canonical Wnt signaling via LRP5, thus promoting osteogenic differentiation and hindering stem cell proliferation [81]. Together with these results, our findings suggest that N-cadherin signaling, and potentially its effect on β-catenin may be a regulatory switch between MSC fate potential and proliferation. Interestingly, the cross talk between N-cadherin, canonical Wnts and β-catenin signaling might also involve RhoA activation due to Wnt5a signaling. The cytoplasmic domain of N-cadherin is linked with either β-catenin or plakoglobin (γ-catenin), which bind through α-catenin to anchor to actin filaments [62]. RhoA-induced actin fibril tension may result in conformational alterations in adherens junction proteins, leading to β-catenin mobilization [82]. It has been demonstrated that RhoA activation and the resulting tension within the actin cytoskeleton play a key roles in guiding osteogenic lineage commitment while inhibiting both adipogenic and chondrogenic differentiation [64]–[66], [83]. However, recent studies have also linked anther small GTPase, Rac1, in cell fate commitment [83], [84]. Specifically, Woods and colleagues have demonstrated that while RhoA activation inhibits chondrogenic differentiation, Rac1 promotes it, and this mechanism involves increased expression of N-cadherin as well as increased cell-cell contacts in cellular condensations [84]. Additionally, it was shown that Rac1 also plays a potent role in canonical Wnt signaling and normal limb bud development [83]. Interestingly, this may indicate that RhoA plays a role in inhibiting N-cadherin association with β-catenin and initiating osteogenic lineage commitment, however further work must be conducted to understand if this hypothesis is valid.

In summary, we determined that the mechanical microenvironment that MSCs are exposed to upon migration into bone may initiate multiple intracellular signaling pathways required for osteogenic lineage commitment. Specifically, flow weakens N-cadherin association with β-catenin enabling β-catenin to translocate to the nucleus and initiate gene transcription, which is necessary for flow induced Runx2 expression. Furthermore, oscillatory fluid flow additionally up-regulates Wnt5a and Ror2, both of which are necessary for flow induced RhoA activation. This flow-induced Wnt5a signaling and RhoA activation are necessary for osteogenic differentiation due to flow [23]. Interestingly, both Wnt5a with Ror2 as well as N-cadherin have a demonstrated potential to inhibit canonical Wnt signaling suggesting that these proteins may be involved in a β-catenin signaling pathway that differs from canonical Wnt induced β-catenin nuclear translocation. With the recent findings suggesting cross-talk between small GTPase proteins, adherens junctions and Wnt signaling, further work must be conducted to understand the relationship or cross talk between these pathways that ultimately result in the converged regulation of Runx2.
